# Multi-stage structure-based virtual screening approach towards identification of potential SARS-CoV-2 NSP13 helicase inhibitors 

**DOI:** 10.1080/14756366.2021.2022659

**Published:** 2022-01-10

**Authors:** Mahmoud A. El Hassab, Wagdy M. Eldehna, Sara T. Al-Rashood, Amal Alharbi, Razan O. Eskandrani, Hamad M. Alkahtani, Eslam B. Elkaeed, Sahar M. Abou-Seri

**Affiliations:** a Faculty of Pharmacy, Department of Pharmaceutical Chemistry, King Salman International University (KSIU), Ras Sudr, Egypt; b Faculty of Pharmacy, Department of Pharmaceutical Chemistry, Kafrelsheikh University, Kafrelsheikh, Egypt; c Department of Pharmaceutical Chemistry, College of Pharmacy, King Saud University, Riyadh, Saudi Arabia; d Department of Pharmaceutical Sciences, College of Pharmacy, AlMaarefa University, Riyadh, Saudi Arabia; e Faculty of Pharmacy, Department of Pharmaceutical Chemistry, Cairo University, Cairo, Egypt

**Keywords:** SARS CoV-2 NSP13 helicase, protein-ligand interaction fingerprint, structure-based pharmacophore, docking, molecular dynamics simulations

## Abstract

On account of its crucial role in the virus life cycle, SARS-COV-2 NSP13 helicase enzyme was exploited as a promising target to identify a novel potential inhibitor using multi-stage structure-based drug discovery approaches. Firstly, a 3D pharmacophore was generated based on the collected data from a protein-ligand interaction fingerprint (PLIF) study using key interactions between co-crystallised fragments and the NSP13 helicase active site. The ZINC database was screened through the generated 3D-pharmacophore retrieving 13 potential hits. All the retrieved hits exceeded the benchmark score of the co-crystallised fragments at the molecular docking step and the best five-hit compounds were selected for further analysis. Finally, a combination between molecular dynamics simulations and MM-PBSA based binding free energy calculations was conducted on the best hit (compound **FWM-1**) bound to NSP13 helicase enzyme, which identified **FWM-1** as a potential potent NSP13 helicase inhibitor with binding free energy equals −328.6 ± 9.2 kcal/mol.

## Graphical Abstract

**Figure:**
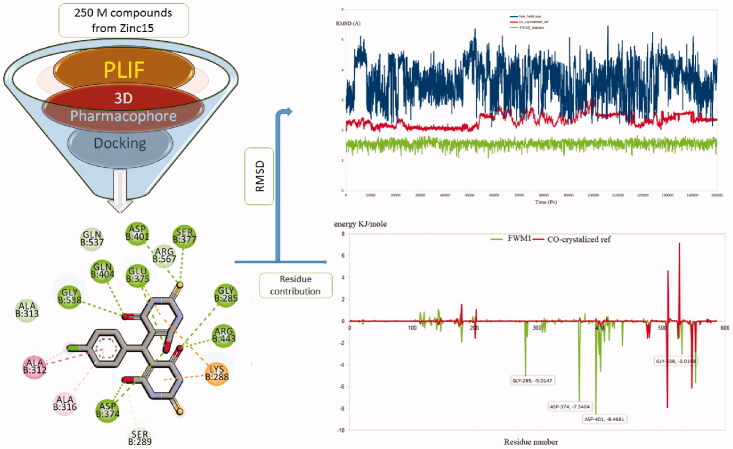

## 1. Introduction

Two years ago, the world was struck by a rapidly growing respiratory infection caused by the severe acute respiratory syndrome-related coronavirus 2 (SARS-CoV-2). Due to the rapid spread of the infection in over 200 countries, the World Health Organisation (WHO) had to declare the infection as a pandemic disease in March 2020^1^. Till now, the virus had infected more than 243 million and killed nearly 4.9 million. The restricted safety measures taken by most of the world countries are not enough especially to prevent the successive waves of infections from the COVID-19. Apart from Remdisvir, the FDA did not approve any specific drugs for COVID-19. Unfortunately, Remdisvir itself failed to demonstrate satisfying results in decreasing the mortality levels and preventing infection associated-dyspnea^2–4^. Better outcomes were obtained from the recently approved vaccines, however, reaching the normal conditions is still outreached and the number of infections is still growing, especially with the emergence of new virus variants that could be more resistant to vaccines. Accordingly, there is an urgent need to rapidly develop a specific effective treatment for the COVID-19 infection.

Scientific efforts identified many enzymes with crucial roles in the life cycle of SARS-CoV-2 virus. These enzymes are namely, RNA-dependent RNA polymerase, papin-like protease, spike glycoprotein, helicase, methyltransferase, and the main protease (Mpro)^5^. Targeting any of these enzymes represents an effective method to identify potential therapy for COVID-19 infection.

Among the above-mentioned targets, the helicase enzyme plays an essential role in the viral RNA replication in concert with the replication-transcription complex (NSP7/NSP8/NSP12)^6^. It has two reported functions, firstly it catalyses the unwinding of viral double-stranded RNA in a 5′ to 3′ direction, and secondly, it has a vital role in the formation of the viral 5′ mRNA cap^7^^,^^8^. Despite its crucial role, the helicase enzyme stands as an under-represented target, taking less attention than both RNA-dependent RNA polymerase and the Mpro. Nevertheless, targeting the helicase enzyme could open a new horizon for the combined therapy against SARS-CoV-2 virus that could easily overcome any emerged resistance. Moreover, the helicase enzyme is highly conserved amongst different Coronavirus strains as evidenced by a single amino acid mutation between SARS COV and SARS-CoV-2^9^. Thus, SARS-CoV-2 helicase inhibitors could serve as an effective broad-spectrum antiviral against current and future COVID infections^9^. To this end, our team was triggered to implement computational approaches to accelerate the drug discovery of SARS-CoV-2 helicase inhibitors.

**Figure 1.**
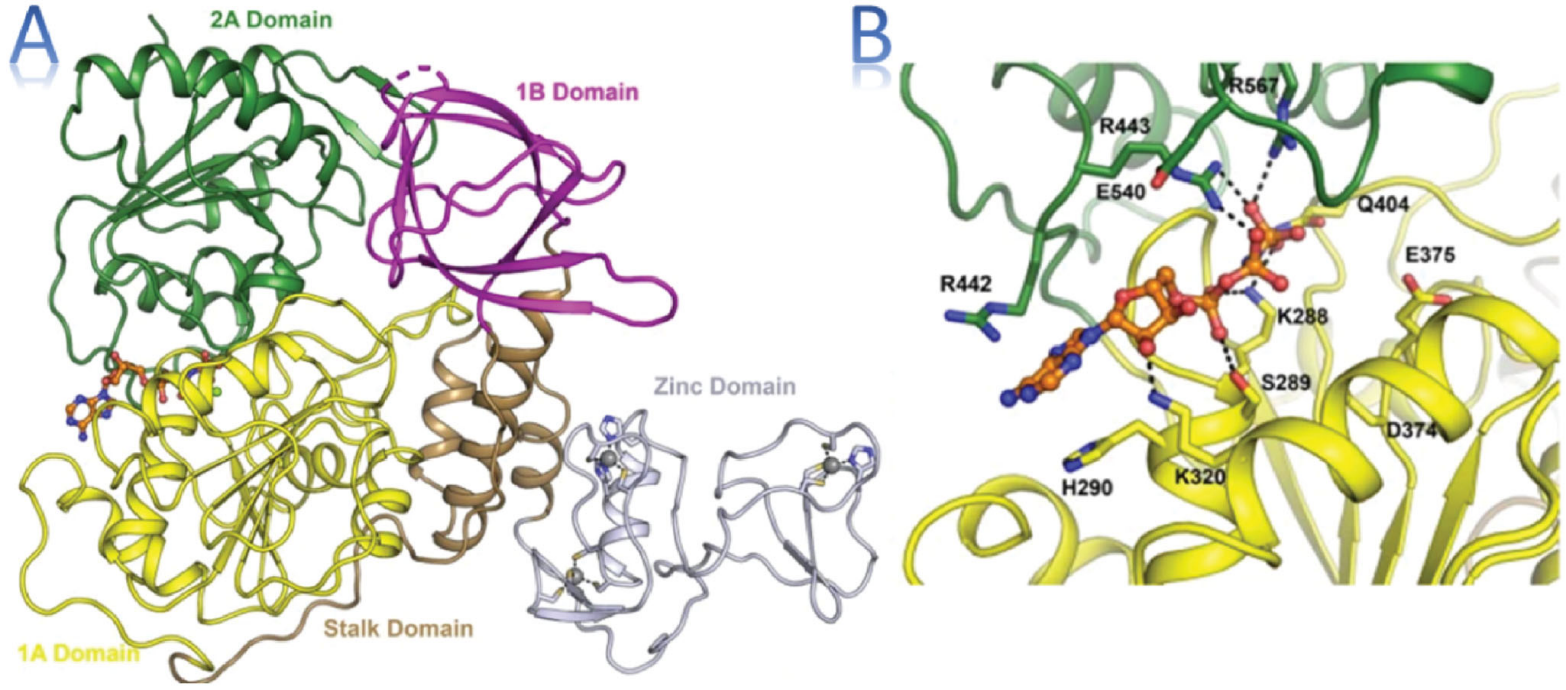
(A) Structure overview of SARS-CoV-2 helicase with domains labelled and coloured individually, (B) Close view of the AMP binding form using the same colour^9^.

The crystal structure of the SARS-CoV-2 helicase has been reported by many researchers making the way for computer-aided drug design studies. Pan-Dataset Density Analysis (PanDDA) study has reported the X-ray crystallographic structure of the SARS-CoV-2 helicase bound to various fragments^9^. The solved structure of the enzyme contains 5 domains, an N-terminal Zinc binding domain (ZBD), a helical “stalk” domain, a beta-barrel 1B domain, and two “RecA like” helicase subdomains 1A and 2A that contain the residues responsible for nucleotide binding and hydrolysis Figure 1. In this work, we aimed to apply a multi-stage virtual screening approach including 3D pharmacophore, molecular docking, molecular dynamics, and MMPBSA calculations to screen the ZINC 15 database searching for a new potential inhibitor for the helicase enzyme.

## 2. Materials and methods

### 2.1. PLIF and 3D pharmacophore

Five crystal structures of SARS-COV-2 helicase in complex with five different fragment inhibitors PDB IDs: 5RL9, 5RLJ, 5RLN, 5RLO and 5RLW were acquired from PDB and used for PLIF study. MOE 2019 was implemented to generate the PLIF data using its default PLIF generation wizard. Initially, all the proteins were prepared using the in-built QuickPrep wizard available in the MOE suite^10^. Then, all five entries were superimposed forming a consensus binding site containing the five fragments. Finally, the PLIF wizard was implemented to prepare and generate the interaction data. The query generator tool panel in the MOE was employed to construct the 3D pharmacophore by converting the gather data into their actual 3D coordinates. In the query generator wizard, the featured coverage was set to 20% to make efficient use of the whole binding site. Finally, Zinc database was screened using the pharmacophore to find prospective SARS-CoV-2 helicase inhibitors.

### 2.2. Database generation for pharmacophore screening

Zinc Drug-like database with 250 Million compounds (available at http://zinc.docking.org) was downloaded and converted into a single database file with extension .mdb by MOE suite (MOE, 2019. https://www.chemcomp.com). Energy minimisation of the database was conducted under AMBER12: EHT force field^10^.

### 2.3. Pharmacophore-based virtual screening

The prepared database was screened through the generated pharmacophore model using MOE pharmacophore software to match all the features in the pharmacophore hypothesis. The tolerating distance was designated to 2.0 Å and a fitness score was used to rank the database hits based on their RMSD with the hypothesis involving site matching, vector alignments, and volume terms. Compounds that passed the pharmacophore filter were further evaluated through docking-based virtual screening.

### 2.4. Docking-based virtual screening

A rough validation procedure was performed and RMSD was calculated between the co-crystallised ligand and re docked poses for all the PDB IDs used in the PLIF study. The best co-crystallised reference and the hits obtained from the pharmacophore were docked into the 5RLW using Vina Autodock software after preparing the protein and ligands in the pdbqt format^11^. The binding site was determined from the binding of the co-crystallised reference using the grid box function in the M.G.L 1.5.6 tools. The docking results were then analysed using a discovery studio visualiser to generate 2D and 3D for the selected hits.

### 2.5. Molecular dynamics (MD)

#### 2.5.1. MD production

To conduct the required molecular dynamics (MD) simulations, Groningen Machine for Chemical Simulations GROMACS 5.1.1 software was employed^12^. To validate the retrieved virtual screening results, three MD simulation experiments were conducted. Two simulation experiments were performed on the enzyme in complex with the **FWM-1** and the crystal reference. And in the third one was performed using the free enzyme a lone. GROMOS96 force field was implemented to generate the ligand topologies using the GlycoBioChem PRODRG2 Server^13^. Later on, each of the generated ligand topologies was joined with the enzyme topology to generate the complex topology. As previously published by our group, the typical scheme for enzyme–ligand simulations by GROMACS was applied, starting with system solvation using single point charge (SPC) water model and ending with neutralisation by adding the suitable number of counter-ions^14–17^.

The three solvated neutralised systems were energy minimised under GROMOS96 43a1 force field using the steepest descent minimisation algorithm with a maximum of 50,000 steps and <10.0 kJ/mol force under. All the systems were equilibrated to the used temperature (310 K) and pressure (1 atm) using NPT ensemble for 8 ns preceded by NVT ensemble for 2 ns. To compute the long-range electrostatic values, the particle mesh Ewald (PME) method with a 12 Å cut-off and 12 Å Fourier spacing was implemented. All The systems were subjected to a production stage of 150 ns. Every two consecutive steps were separated by 2 fs and the structural coordinates were saved every 30 ps. V-rescale weak coupling method (modified Berendsen thermostat) and Parrinello–Rahman method were used to regulate the temperature (310 K) and the pressure (1 atm) throughout the simulation^18^^,^^19^.

The root means square deviation (RMSD) and root means square fluctuation (RMSF) of the entire system and each residue respectively, were calculated from the generated trajectories from the production step. To further analyse the predicted binding mode of **FWM-1**, various scripts of GROMACS were used to calculate the distances of the formed hydrogen bonds between the **FWM-1**and the NSP13 helicase enzyme.

#### 2.5.2. MM-PBSA calculation and per residue contribution

To compute the binding-free energy calculations using the MM–PBSA approach, the following equation was employed (further details are provided in the Supporting information):

$${\Delta G}_{\left(\mathrm{Binding}\right)}=G_{\left(\mathrm{Complex}\right)}-G_{\left(\mathrm{Receptor}\right)}-G_{\left(\mathrm{Ligand}\right)}$$

These calculations were done for the two complexes of helicase–**FWM-1** and helicase–crystal reference using the g-mmpbsa package^20^. The energy contribution of each residue was calculated by decomposition of the total free energy per residue.

## 3. Results and discussion

### 3.1. PLIF and 3D pharmacophore

The protein-ligand interaction fingerprint (PLIF) employs a small database of active ligands or fragments bound to the same target within the same binding site^21^. The technique aims to elucidate and establish the most stable and crucial interactions accounting for the ligands activities^22^. These interactions can be further utilised in computational chemistry studies including virtual screening protocols aiming for novel drug discovery. The generation of a 3D pharmacophore is one of the most important protocols resulting from the analysis of the PLIF interactions. Furthermore, the generated 3D pharmacophore could be implemented as a screening filter to fetch active compounds against enormous databases usually in millions^23^^,^^24^.

**Figure 2.**
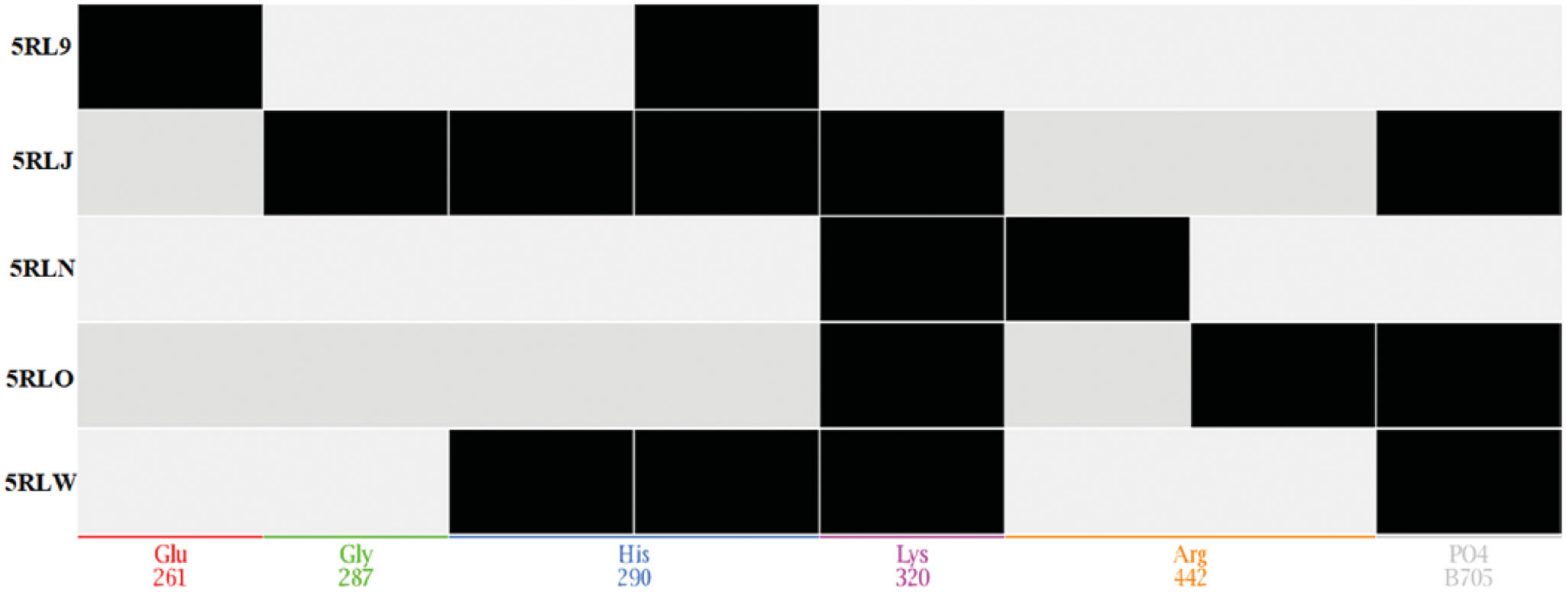
PLIF interaction matrix.

**Figure 3.**
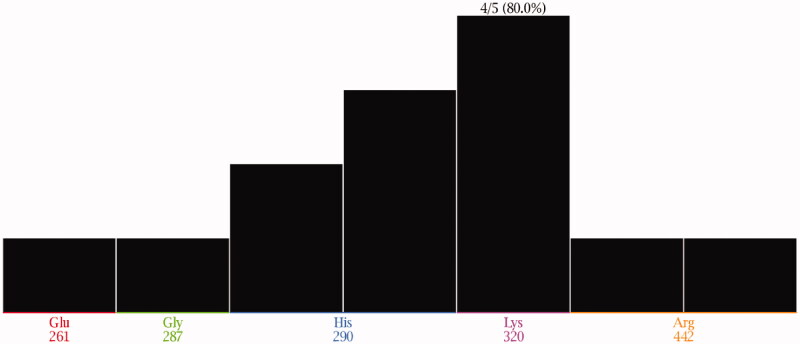
PLIF interaction histogram.

In the current study, five crystal structures for SARS-COV-2 helicase in complex with five different fragment inhibitors discovered from PanDDA study were used (PDB IDs: 5RL9, 5RLJ, 5RLN, 5RLO, and 5RLW). Initially, the five crystal structures were prepared and aligned showing the five fragments fitting to different sub-pockets in the same binding site. In Figure 2, a representational matrix of each interaction is a vertical column on the corresponding residue with a black square indicating the presence of a specific interaction. Figure 3 is a histogram with *X*-axis as amino acid residues and *Y*-axis displays black bars representing the frequency of occurrence amongst crystal structures.

**Figure 4.**
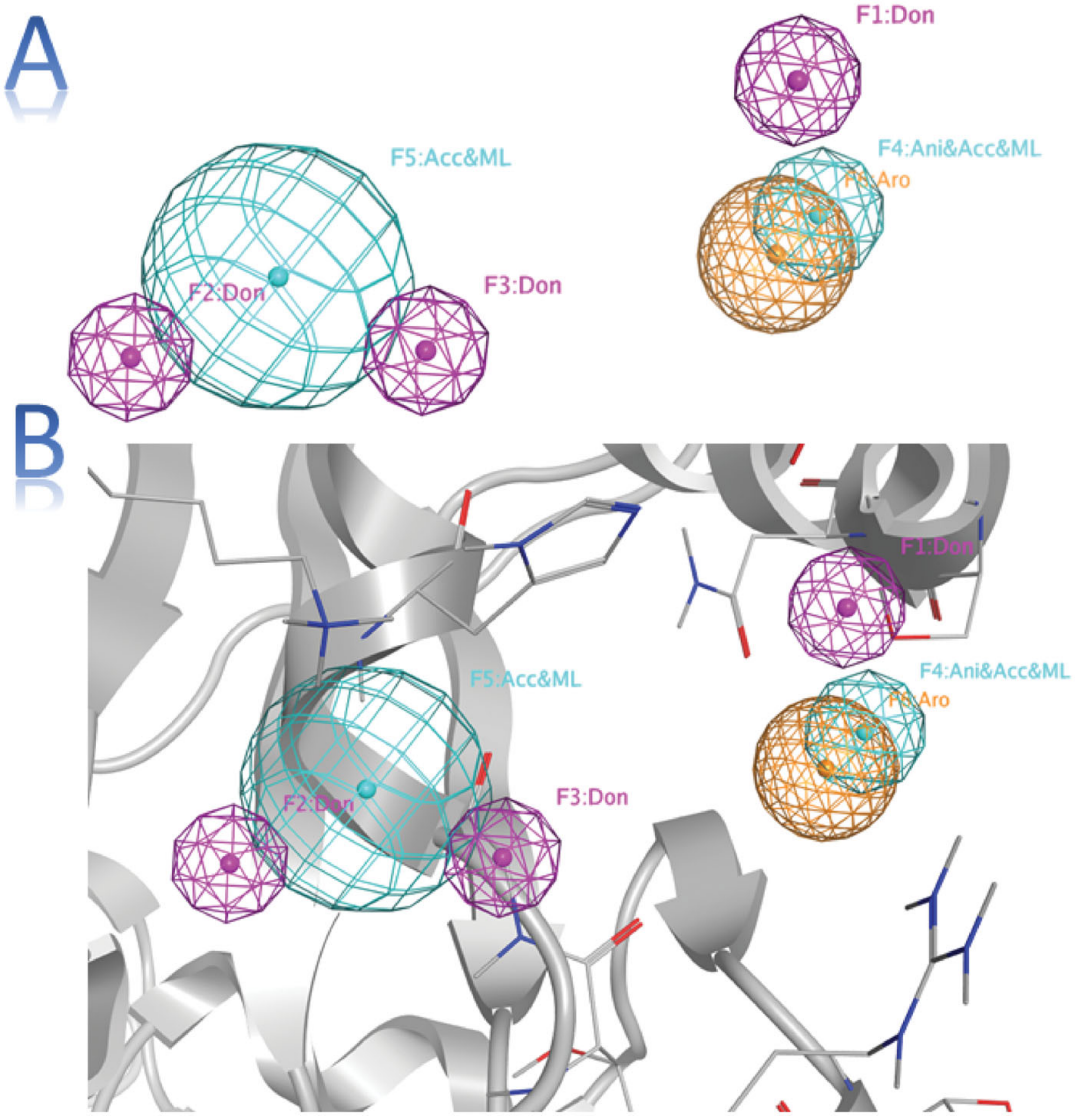
(A) The generated 3D pharmacophore showing three pharmacophoric features: pink (H-bond donor), cyan (H-bond acceptor) and orange (aromatic centre). (B) The generated 3D pharmacophore within the pocket.

Upon inspection of the PLIF outcomes, five amino acid residues (Glu261, Gly287, His290, Lys320, and Arg442) exhibited the most stable interactions. Closer insights revealed that no specific residue was involved in the interaction with all the studied fragments Figure 3. Finally, the PLIF data were used to generate a structure-based 3D pharmacophore to discover potential helicase inhibitors. The pharmacophore is composed of three types of features namely; hydrogen donor, hydrogen acceptor, and an aromatic centre. The three pharmacophoric features were distributed across six components; three hydrogen donors, two hydrogen acceptors, and one aromatic centre Figure 4.

The generated pharmacophore was used to filter 250 million compounds from ZINC 15 database^25^. Only 13 compounds conferred all the pharmacophoric features and were then evaluated against SARS-CoV-2 helicase enzyme through a molecular docking study.

### 3.2. Docking studies

Computer-aided drug design provides various tools to aid and shorten drug discovery costs and time. Among those tools is molecular docking which predicts interactions between proposed ligands and targets. Additionally, analysis of these interactions nudges futuristic optimisation and development of proposed ligands^26^.

**Figure 5.**
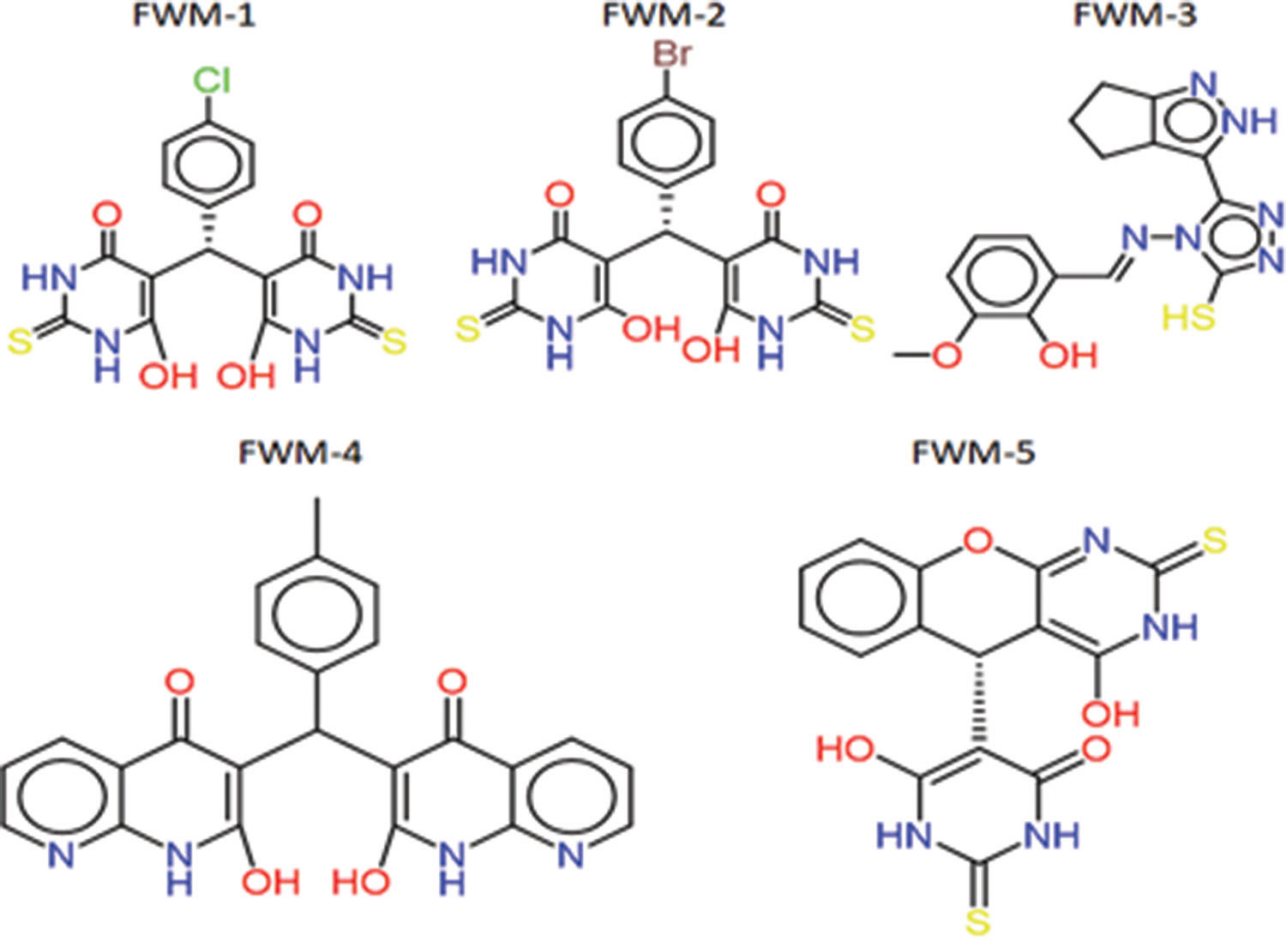
2D Structures of the five generated hit compounds.

**Figure 6.**
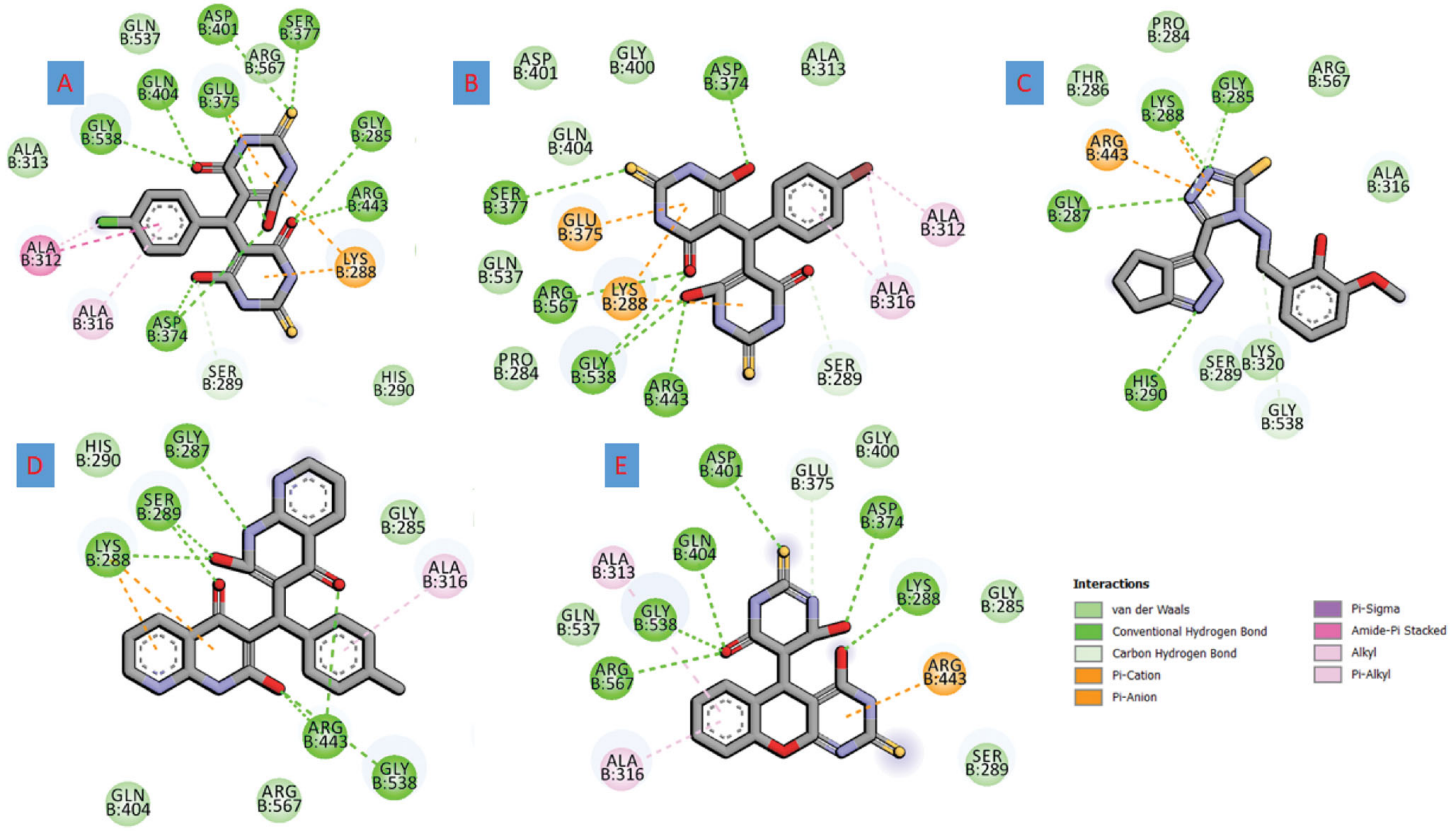
2D Presentation of the docked hits **FWM1-5** [(A–E), respectively] into helicase active showing important interactions in dotted lines.

The resulting 13 compounds from pharmacophoric search were docked into the active site of SARS-CoV-2 helicase. Before commencing the docking of the hit compounds, a pose retrieval experiment was conducted through the re-docking of the five co-crystallised ligands into their enzyme. All the regenerated ligand-protein structures had RMSD value less than 2 Å compared to the co-crystallised ligand indicating a valid docking protocol. The previous step highlighted the co-crystallised ligand of the PDB ID: 5RLW as the best ligand achieving a binding score of −6.5 Kcal/mole. Subsequently, the 5RLW protein and its co-crystallised ligand were selected for the docking conduction and benchmarking the docking results respectively. All the compounds achieved higher binding affinities than the benchmark fragment (−6.5 Kcal/mole) with the most potential active compounds (**FWM-1, FWM-2, FWM-3, FWM-4**, and **FWM-5**) were selected for further analysis. The 2D structure of the five selected compounds and their interactions are supplied in Figures 5 and 6.

All five compounds exhibit potent binding through multiple types of interactions with helicase. For instance, compound **FWM-1** showed a binding score of −12.4 Kcal/mole and was engaged in many types and numbers of interactions. The retrieved lead compounds fit perfectly within the active site of the helicase forming H-bond with GLY285, ASP374, GLU375, SER377, ASP401, GLN404, ARG443, and GLY538 as well as hydrophobic interactions with ALA312, ALA316, LYS288, SER289, and GLU375. In a similar way, **FWM-2**, **FWM-3**, **FWM-4**, and **FWM-5** performed similar modes of interactions with scores of −10.9, −9.1, −11.1 and −10.2 Kcal/mole respectively as shown in Table 1.

**Table 1.** Binding score and key interactions between the docked hits **FWM1-5** with the helicase enzyme.

Compound	Score (Kcal/mole)	Residues involved in H-bonds Interactions	Residues involved in hydrophobic Interactions
**FWM-1**	−12.4	GLY285, ASP374, GLU375, SER377, ASP401, GLN404, ARG443, and GLY538	ALA312, ALA316, LYS288, SER289, and GLU375
**FWM-2**	−10.9	ASP374, SER377, ARG567, ARG443, and GLY538	ALA312, ALA316, LYS288, SER289, and GLU375
**FWM-3**	−9.1	GLY285, GLY287, LYS288, and HIS290	GLY285, ARG443, and GLY538
**FWM-4**	−11.1	GLY287, LYS288, SER289, ARG443, and GLY538	ALA316 and LYS288
**FWM-5**	−10.2	LYS288, ASP374, ASP401, ARG567, GLN404, and GLY538	ALA313, ALA316, ARG443, and GLU375

**Figure 7.**
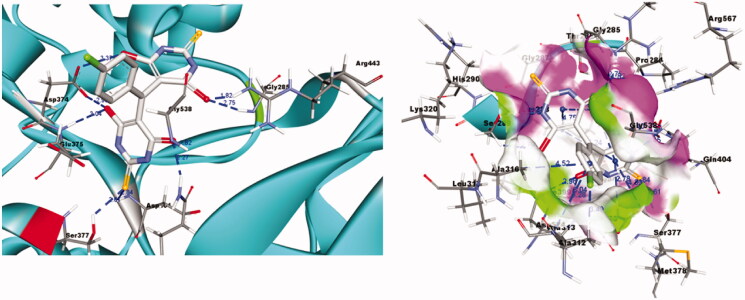
3D Representation for **FWM-1** (thick stick representation) docked into the active site of helicase (ribbon presentation, key interaction amino acids are shown in thin stick representation) showing significant interactions in dotted lines.

Deep insights of the predicted binding of **FWM-1** as depicted in Figure 7 revealed an optimum orientation in the active site of the SARS-COV-2 helicase enzyme. As reported in the literature, the binding site of the SARS COV and similarly SARS-COV-2 helicase is located between the 1 A and 2 A domains. This binding site composes some essential amino acids necessary for the ATP binding; ASP374, GLU375, SER377, ASP401, GLN404, ARG443, LYS288, SER289, ARG567, and GLY538. Interestingly, **FWM-1** was able to fit perfectly in the ATP binding domain, forming hydrogen bonds with GLY285, ASP374, GLU375, GLN404, ARG443, and GLY538. Further stabilisation of the binding pose was ensured through the oxygen and the sulphur of the amide and thiourea, respectively that acted as acceptor groups for the hydrogens of SER377 and ASP401. Moreover, the chlorophenyl group oriented itself the hydrophobic sub-pocket consisting of ALA312, ALA313, and ALA316 to ensure maximum stability through hydrophobic interaction. Worthy mentioning, no co-crystallised fragments were able to bind with the reported amino acids in the ATP binding pocket. This is attributed to the small size of the fragments and their limited function groups. Thus they could only be oriented in a small sub-pocket forming little numbers of interactions. In contrast, the proposed full inhibitors **FWM1-5** have the perfect mix of size, polar and non-polar function groups. Accordingly, the five retrieved hits were able to fill the ATP-binding domain, accommodating various interactions with the necessary amino acids.

In conclusion, **FWM-1** is a promising candidate that would effectively disrupt the binding of ATP to the SARS-COV2 helicase enzyme. This disruption represents a powerful strategy to tackle the ATPase activity of the SARS-COV2 helicase enzyme preventing a cornerstone step in the life cycle of the virus. Accordingly, the retrieved complex between **FWM-1** and SARS-COV2 helicase enzyme was further studied and examined through molecular dynamic simulation and MPBSA calculations.

### 3.3. Molecular dynamics

#### 3.3.1. RMSD and RMSF analysis

**Figure 8.**
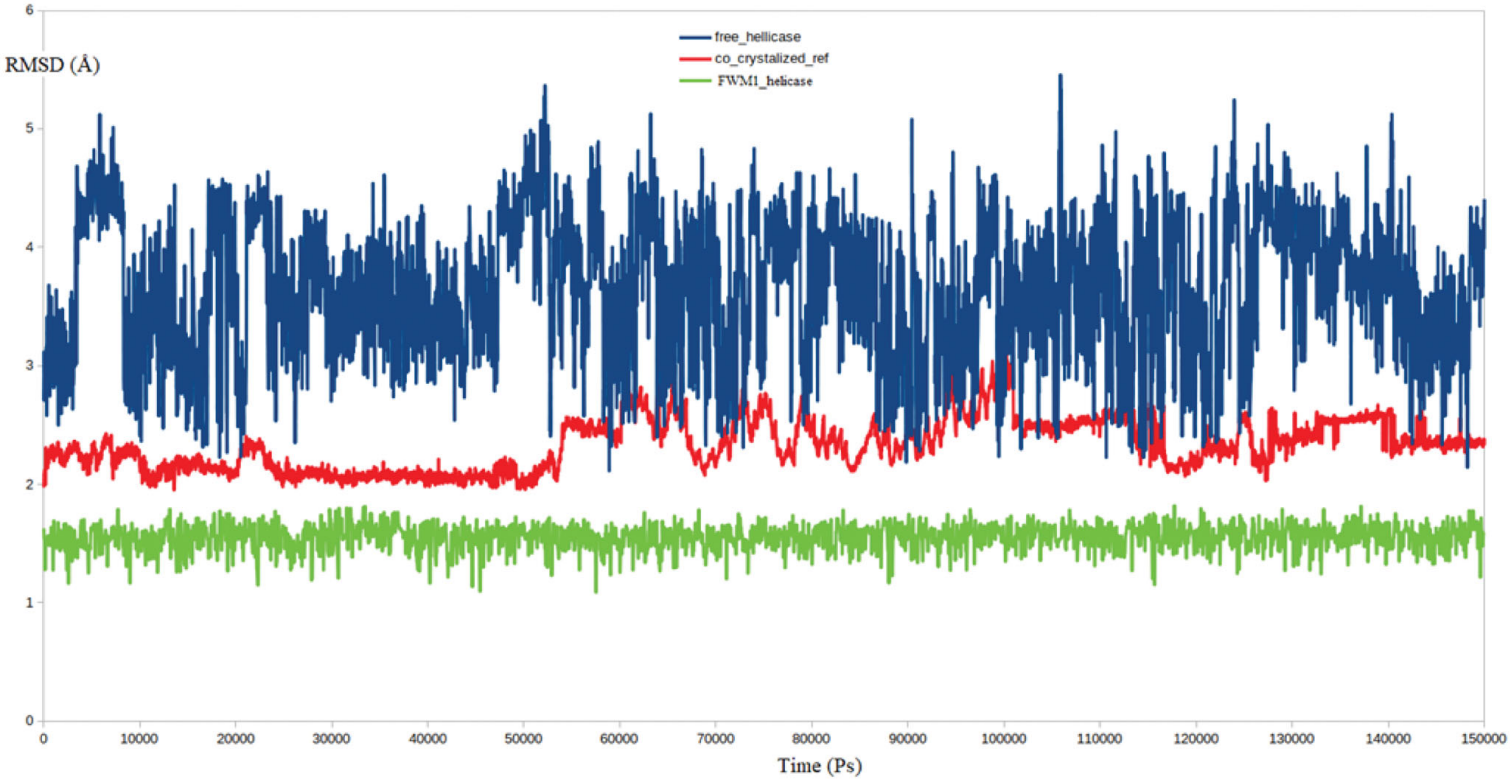
RMSD analysis for the MD simulations for the native enzyme (blue), co-crystallised reference (red) and **FWM-1** (green).

Molecular dynamics (MD) simulation has been an inevitable technique in studies involving *in silico* drug discovery. MD provides many important parameters, data, and figures necessary in various computational and molecular modelling studies. One of the most common applications of the MD is the precise determination of the binding strength between a ligand and its target. Other applications are well reported such as studying macromolecules’ nature and characterising the effect of certain mutations on the resistance profile of many drugs^27^. Therefore, it was logistic to take the advantage of the MD to further endorse our protocol of virtual screening approach. Three MD simulation experiments were conducted on the free helicase, co-crystallised-helicase and **FWM**-**1-**helicase. Interestingly, the calculated RMSD for the free helicase reached more than 5 Å, while the RMSD of the co-crystalised ligand and **FWM**-**1-**helicase complexes reached 2.95 and 1.6 Å respectively at their maximum deviations Figure 8. The ability of **FWM-1** to produce such a lower RMSD value is a powerful indicator of its ability to restrict the dynamic flexible nature of the helicase through the formation of a stable complex.

**Figure 9.**
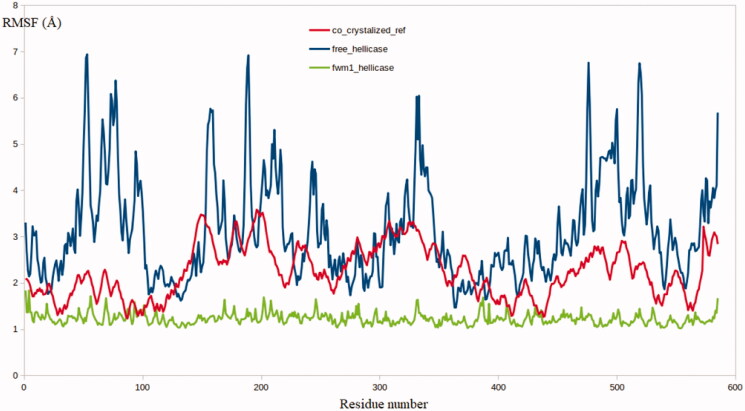
The RMSF analysis for the MD simulations for the native enzyme (blue), co-crystallised reference (red) and **FWM-1** (green).

As expected, the obtained results from the RMSF calculations for all the residues in the three systems were highly matched to the RMSD results. The **FWM**-**1-**helicase complex showed a significantly lower level of residues fluctuation compared to the co-crystallised reference and the free enzyme Figure 9. Worth noting that, the high flexibility of SARS-COV2 helicase as depicted from the high values of RMSD and RMSF is consistent with its function to process the virus genome as a core step in the virus replication cycle. Given account to the superiority of **FWM-1** to restrain the movement of the helicase enzyme, it is predicted to inhibit the enzyme at highly potent levels.

**Figure 10.**
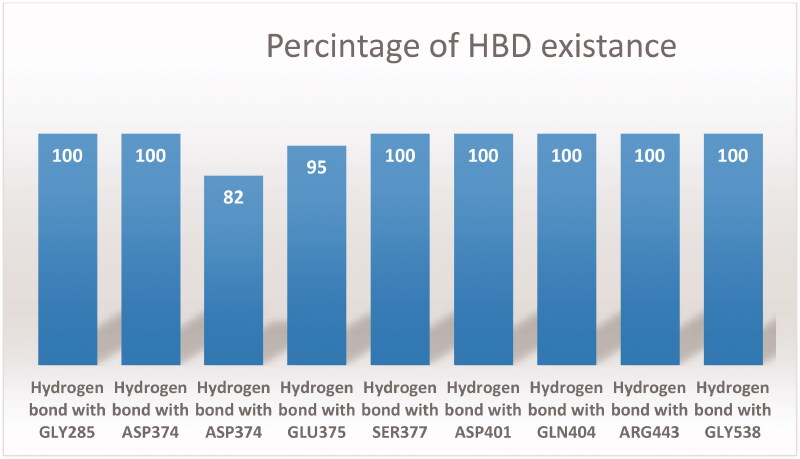
The percentage of existence for each formed Hydrogen bond between **FWM-1** and the helicase.

Further inspection of the stability of **FWM**-**1**-helicase complex was conducted by measuring the average distance, as well as, the standard error for each hydrogen bond formed between **FWM-1**and its target. It is well reported that a hydrogen bond is considered valid only if the distance between the hydrogen bond donor and acceptor is maintained at less than 3.5 Å. As shown in Table 2 and Figure 10, **FWM-1** was able to fulfil the previous criterion for all its hydrogen bond interactions through the entire production phase of the MD simulation.

**Table 2.** The average distances of all the hydrogen bonds formed between **FWM-1** and SARS-COV-2 helicase through the entire 150 ns MD simulation.

Hydrogen bond name	Average distance (Å) ± SD
Hydrogen bond with GLY285	2.71 ± 0.06
Hydrogen bond with ASP374	2.88 ± 0.09
Hydrogen bond with ASP374	3.25 ± 0.32
Hydrogen bond with GLU375	3.15 ± 0.36
Hydrogen bond with SER377	2.49 ± 0.17
Hydrogen bond with ASP401	2.85 ± 0.02
Hydrogen bond with GLN404	2.31 ± 0.07
Hydrogen bond with ARG443	1.78 ± 0.05
Hydrogen bond with GLY538	2.91 ± 0.13

#### 3.3.2. Binding free energy calculations using MM-PBSA approach

The binding free energies between **FWM-1** and co-crystallised reference with SARS COV-2 helicase were calculated from all the conformations in the saved trajectories using the MM-PBSA approach. The g_mmpbsa package generated by Kumari et al. ^20^, was used to calculate all the MM-PBSA binding free energy forms (van der Waal energy, Electrostatic energy, Polar solvation energy, and SASA energy) for the two complexes of SARS-COV-2 helicase with **FWM-1** and co-crystallised reference (Table 3). The calculated binding energy shows a significantly higher affinity for **FWM-1** compared to co-crystallised reference which is consistent with the results from the docking and MD calculations.

**Table 3.** The binding free energy and the interaction energies for both **FWM-1** and co-crystallised complexes.

Complex	Δ*E*_binding_ (kJ/mol)	Δ*E*_electrostatic_ (kJ/mol)	Δ*E*_Vander Waal_ (kJ/mol)	Δ*E*_polar solvation_ (kJ/mol)	SASA (kJ/mol)
**FWM-1**	−328.6 ± 9.2	−157.7 ± 8.9	−214.6 ± 11.8	72.4 ± 6.3	−28.7.1 ± 1.6
Co-crystallised reference	−159.7 ± 6.7	−86.2 ± 5.4	−127.6 ± 8.2	75.3 ± 6.1	−21.2 ± 1.1

**Figure 11.**
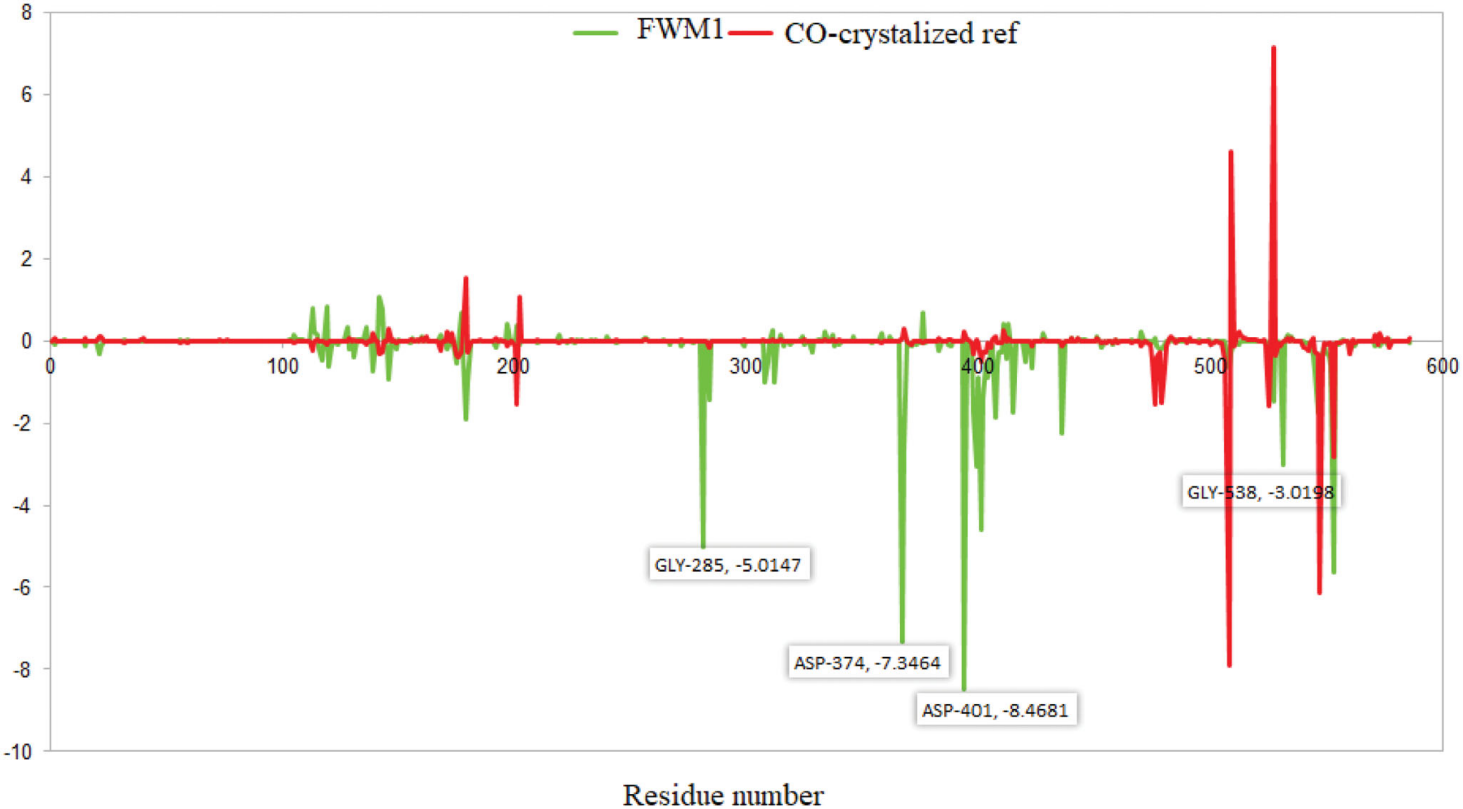
The per residue contribution for the binding free energy for **FWM-1** (green) and co-crystallised ligand (red).

Further insights into the binding between **FWM**-**1** and the helicase enzyme were provided via the calculation of every residue contribution in terms of binding free energy. This contribution was calculated by decomposing the total binding free energy of the system into per residue contribution energy, Figure 11. As depicted from Figure 11, all the Key interacting amino acids GLY285, ASP374, GLU375, SER377, ASP401, GLN404, ARG443, and GLY538 showed favourable significant contribution to binding free energy. In agreement with docking, MD, and MMPBSA results, the results of per residue decomposition predicted the great potentiality for **FWM-1** to produce a strong stable complex with the helicase enzyme.

## 4. Future prospects

In this work, we aimed at both the identification of potential inhibitors for SARS-CoV-2 NSP13 helicase enzyme as well as the establishment of a valid virtual screening approach for further COVID-19 drug discovery. Interestingly, our team has identified certain required pharmacophoric features essential for the design of potential SARS-CoV-2 NSP13 helicase enzyme inhibitors. The promising results shown by FWM-1, as a lead candidate, encourage further experimental studies to biologically evaluate its potential activity and also to provide practical means for further optimisation.

## 5. Conclusions

Since 2019, the world has been in a fierce confrontation with the respiratory pandemic infection of COVID-19 caused by SARS-Cov-2. Till now, the world health organisation reported more than 243 million cases and nearly 4.9 million deaths worldwide. Restoring normal life conditions is still far from reaching even with strict safety measures and massive vaccination programs. Developing specific therapy represents the best mean to accelerate restoring normal human activities and put an end to the successive waves of infection striking many countries. In this work, the SARS-COV-2 NSP13 helicase was selected as an attractive drug target for the development of COVID-19 therapy. In continuance of our team effort, here we report the application of a multi-stage virtual screening approach to identify potential lead compounds against NSP13 helicase enzyme. Firstly, protein-ligand fingerprinting study using the key ligand-target interactions was conducted to facilitate the generation of a Structure-based pharmacophore. 250 million compounds from the ZINC15 molecular database set were virtually screened through the generated 3D pharmacophore. Only 13 compounds with function groups fulfilling all the pharmacophore features were selected for a molecular docking step into the NSP13 active site. All the 13 hits exceeded the docking score benchmark and the highest scoring five compounds were selected for binding mode analysis. MD simulation and binding free energy calculations for the best lead FWM-1, revealed superior binding affinity (−328.6 ± 9.2 Kcal/mole) and stability with NSP13 helicase enzyme. Further inspection of the per-residue energy contribution of the binding site amino acids with FWM-1 revealed a highly favourable contribution of the key amino acids in the ATP-binding domain.

## Supplementary Material

Supplemental Material
