# Supplementary material for: Multi-stage structure-based virtual screening approach towards identification of potential SARS-CoV-2 NSP13 helicase inhibitors
Source: J Enzyme Inhib Med Chem. 2022 Jan 10;37(1):563–72. doi: 10.1080/14756366.2021.2022659 (PMC8757614; doi:10.1080/14756366.2021.2022659)
Supplement: Supplemental Material [file IENZ_A_2022659_SM3204.pdf]

Supporting Information

MMPBSA calculations

$$\Delta G_{(Binding)} = G_{(Complex)} - G_{(Receptor)} - G_{(Ligand)}$$

Where $G_{(Complex)}$ is the total free energy of the protein−ligand complex and $G_{(Receptor)}$ and $G_{(Ligand)}$ are the total free energies of the isolated protein and ligand in solvent, respectively. The total free energy of any of the three mentioned entities (complex, receptor and ligand) were calculated for all MD trajectories from its molecular mechanics potential energy plus the energy of the solvation, using the g_mmpbsa package implemented in the GROMACS software. Individual energies along with the values of standard deviations were calculated and then summed together to yield the average total free energy of each component. Finally, to calculate the binding-free energy, the total free energy of the receptor and the ligand were subtracted from the total free energy of the complex.
